# Restorative dentistry and oral rehabilitation: United Kingdom National Multidisciplinary Guidelines

**DOI:** 10.1017/S0022215116000414

**Published:** 2016-05

**Authors:** C Butterworth, L McCaul, C Barclay

**Affiliations:** 1 Merseyside Regional Head and Neck Cancer Centre, University Hospital Aintree and Liverpool University Dental Hospital, Liverpool, UK; 2 Division of Surgery and Anaesthesia, Bradford Teaching Hospitals NHS Foundation Trust, Bradford, UK; 3 University Dental Hospital of Manchester, Manchester, UK

## Abstract

This is the official guideline endorsed by the specialty associations involved in the care of head and neck cancer patients in the UK and provides recommendations on the pre-treatment oral and dental assessment, during and after treatment and oral rehabilitation. Restorative dentists are core members of the multidisciplinary team treating head and neck cancer patients, involved from the treatment planning phase through to long-term rehabilitation.

**Recommendations:**

• Preventative oral care must be delivered to patients whose cancer treatment will affect the oral cavity, jaws, salivary glands and oral accessibility. (G)

• Close working and communication between the surgeons, oncologists and restorative dental specialists is important in ensuring optimal oral health outcomes. (G)

• Intensity-modulated radiotherapy has been shown to reduce long-term xerostomia and should be offered to all appropriate patients. (R)

• If patients are deemed at risk of trismus they should be warned and its progressive and potentially irreversible nature explained. (G)

• Where it is known that adjuvant radiotherapy will be given, extractions should take place at primary surgery to maximise the time for healing and minimise the number of surgical events for patients. (G)

• Osseointegrated implants should be considered for all patients having resection for head and neck cancer. (G)

## Introduction

The consultant in restorative dentistry and oral rehabilitation is a core member within the head and neck cancer team as many patients face complex oral rehabilitation and dental health issues during and after their treatment. This section addresses the issues relating to pre-treatment oral and dental assessment, preventative advice, during and after treatment and oral rehabilitation.

## Oral and dental assessment prior to primary treatment

Patients whose oral cavity, teeth, salivary glands and jaws will be affected should have assessment and appropriate management as early as possible to allow time for any necessary dental treatment.^1^ This should render patients dentally fit before treatment and ensure the oral cavity can be maintained and rehabilitated after treatment.^2^

The aims of pre-treatment assessment are:

• Avoidance of unscheduled interruptions to primary treatment as a result of dental problems
• Pre-prosthetic planning and treatment, e.g. planning for primary implants and/or impressions for obturator
• Planning for extraction of teeth which are of doubtful prognosis or are at risk of dental disease in the future and are in an area where there would be risk of osteoradionecrosis.^2^ Extractions to be carried out as early as possible in the patient journey but, as a minimum, at least 10 days prior to radiotherapy
• Planning for restoration of remaining teeth as required
• Preventive advice and treatment
• Assess potential for post-treatment access difficulties, e.g. trismus, microstomia.

## Treatment side effects

Treatment for head and neck cancer may involve surgery, chemotherapy and radiotherapy which can cause adverse short- and long-term oral side effects as follows:

Short term:

• Mucositis: inflammation and ulceration of the mucosal lining of the oral cavity
• Infection: chemotherapy-induced neutropenia makes the patient susceptible to bacterial, viral and fungal infections. Oral candidal infections are extremely common following chemotherapy or radiotherapy
• Xerostomia: dry mouth resulting from a decrease in the production of saliva as a result of radiotherapy.

Long term:

• Altered anatomy: surgical ablation and reconstruction can cause permanent changes in oral anatomy making prosthetic rehabilitation difficult
• Rampant dental caries: radiogenic dental caries^3^^,^^4^ is thought to be the result of reduced salivary flow as well as possible direct radiogenic damage to the amelo-dentinal junction by radiotherapy
• Trismus: may be caused by surgical scarring or by radiotherapy induced fibrosis of the masticatory muscles
• Mastication difficulties: if a significant number of opposing pairs of teeth are lost
• Osteoradionecrosis: hypovascularity and necrosis of bone followed by trauma-induced or spontaneous mucosal breakdown, leading to a non-healing wound
• Xerostomia: intensity-modulated radiotherapy (IMRT) reduces the risk of xerostomia after treatment and possibly osteoradionecrosis.^5^

## Management

### Preventive management

• Maintenance of good oral hygiene by effective tooth brushing; flossing daily
• Dietary advice with regard to caries prevention
• Daily topical fluoride application (5000 ppm fluoride toothpaste) in custom-made trays or brush-on.^6^ Daily fluoride mouth rinse, remineralising agents
• Daily use of GC Tooth Mousse^™^ containing free calcium or other remineralising agent^7^
• Saliva replacement therapy and use of frequent saline rinses
• Jaw exercises to reduce trismus.

### Peri-treatment and post-treatment management

#### Oral mucositis and ulceration

Treatments include Chinese medicines, hydrolytic enzymes, ice chips, benzydamine, calcium phosphate, etoposide bolus, manuka honey, iseganan and zinc sulphate.^8^ All have been shown to demonstrate some level of benefit although the response seems to be patient specific. Benzydamine mouthwash has been recommended for those patients receiving moderate radiation without concomitant chemotherapy. The use of amifostine in this setting has now been refuted.

#### Oral candidal infections

There is strong evidence that some antifungal drugs prevent oral candidiasis caused by cancer treatment, but nystatin does not appear to work. Chlorhexidine gluconate has antifungal and antibacterial properties in addition to antiplaque effects; however, its value is still unconfirmed. Its tendency to stain teeth and its alcohol content, which can irritate inflamed tissues, are potential drawbacks.

#### Xerostomia

This can be managed by sipping sugarless fluids frequently, chewing sugarless gum or lozenges, and using a carboxymethyl cellulose saliva substitute as a mouthwash. Oral balance gel may be best accepted by patients because of its extended duration of effect. Acidic salivary stimulants such as Glandosane^™^ should not be used by dentate patients as their pH is below the critical pH of 5.5. Pilocarpine (5–10 mg/day) may improve radiation induced xerostomia in patients with evidence of some intact salivary function.

#### Altered anatomy

Prostheses may be required to replace missing oral and facial tissues. These may be implant supported.

#### Rampant dental caries

Management must be individualised, and patients must be assessed at regular intervals to determine the caries risk and caries activity to provide guidance for maintenance of the dentition.

#### Mastication difficulties

This can be minimised by maintenance of the dentition and use of well-made prostheses.

#### Trismus

Jaw exercises and the use of devices such as the Therabite^™^ prior to and during radiotherapy may limit the severity of trismus, but they will not mobilise fibrosis once it has occurred. They may help surgically induced trismus (as may coronoidectomy). Dental work that was deferred during radiotherapy should be completed. Frequent dental follow-up appointments (3–4 monthly), either with local general or community dental practitioner is warranted for these patients.

#### Oral rehabilitation using osseointegrated implants

Osseointegrated implants allow effective oral and facial rehabilitation following cancer treatment including radiotherapy.^9^^,^^10^ They are used to support oral or facial prostheses.^11^ Appropriate detailed planning and patient selection are important prior to proceeding with treatment. The use of hyperbaric oxygen may be considered prior to elective implant placement in the irradiated jaws (>60 Gy) in an attempt to improve implant survival rates but is a controversial area with currently no clear cut evidence.

**Recommendations**

• Preventive oral care must be delivered to patients whose cancer treatment will affect the oral cavity, jaws, salivary glands and oral accessibility (G)
• Close working and communication between the surgeons, oncologists and restorative dental specialists is important in ensuring optimal oral health outcomes (G)
• IMRT has been shown to reduce long-term xerostomia and should be offered to all appropriate patients (R)
• If patients are deemed at risk of trismus they should be warned and its progressive and potentially irreversible nature explained (G)
• Where it is known that adjuvant radiotherapy will be given, extractions should take place at primary surgery to maximise the time for healing and minimise the number of surgical events for patients (G)
• Osseointegrated implants should be considered for all patients having resection for head and neck cancer (G)

##### Primary dental implants

The placement of intra-oral implants at the same time as tumour resection may be beneficial for carefully selected patients and where there is continuity of the mandible or in patients who require the prosthetic obturation of significant maxillary defects where retention of the obturator is likely to be compromised or in patients undergoing rhinectomy or orbital exenteration.^9^^,^^12^ In patients having segmental resection and reconstruction of the mandible, implant survival and usefulness is improved by delayed placement after suitable prosthodontic planning.^13^

##### Secondary dental implants

For many patients, the placement of osseointegrated implants will be considered following cancer treatment in response to ongoing problems with oral function. A secondary approach allows a detailed assessment of the patient's overall prognosis, their individual risk factors (alcohol, smoking, oral hygiene, radiotherapy, etc.) as well as their anatomical factors such as the presence of reconstructive hard and soft tissue grafts, metal hardware, tongue function and mouth opening. Comprehensive prosthodontic planning should be undertaken prior to surgery and the use of computerised planning and surgical guide stent technology is useful.

### Key points

• Consultants in Restorative Dentistry are core members of the multidisciplinary team dealing with head and neck cancer patients
• Patients whose oral cavity, teeth, salivary glands and jaws will be affected by their treatment should have a dental assessment and appropriate management as early as possible to allow time for any necessary dental treatment
• Patients requiring maxillary obturation should be carefully prepared for treatment by a Restorative specialist who should ideally be present during surgery
• Consideration should be given to the placement of osseointegrated titanium implants at the time of primary resective surgery in selected patients in order to support dental and facial prostheses
• Liaison with the patient's general dental practitioner is important for ongoing dental care with support from the Restorative specialist where advice is required.
